# Psychobiotics: Mechanisms of Action, Evaluation Methods and Effectiveness in Applications with Food Products

**DOI:** 10.3390/nu12123896

**Published:** 2020-12-19

**Authors:** Mariano Del Toro-Barbosa, Alejandra Hurtado-Romero, Luis Eduardo Garcia-Amezquita, Tomás García-Cayuela

**Affiliations:** Tecnologico de Monterrey, Escuela de Ingeniería y Ciencias, Avenida General Ramón Corona 2514, 45138 Zapopan, Jalisco, Mexico; a01229309@itesm.mx (M.D.T.-B.); a01230908@itesm.mx (A.H.-R.); garcia.amezquita@tec.mx (L.E.G.-A.)

**Keywords:** psychobiotic, fermented food, gut-brain-microbiota axis

## Abstract

The gut-brain-microbiota axis consists of a bilateral communication system that enables gut microbes to interact with the brain, and the latter with the gut. Gut bacteria influence behavior, and both depression and anxiety symptoms are directly associated with alterations in the microbiota. Psychobiotics are defined as probiotics that confer mental health benefits to the host when ingested in a particular quantity through interaction with commensal gut bacteria. The action mechanisms by which bacteria exert their psychobiotic potential has not been completely elucidated. However, it has been found that these bacteria provide their benefits mostly through the hypothalamic-pituitary-adrenal (HPA) axis, the immune response and inflammation, and through the production of neurohormones and neurotransmitters. This review aims to explore the different approaches to evaluate the psychobiotic potential of several bacterial strains and fermented products. The reviewed literature suggests that the consumption of psychobiotics could be considered as a viable option to both look after and restore mental health, without undesired secondary effects, and presenting a lower risk of allergies and less dependence compared to psychotropic drugs.

## 1. Introduction

According to the World Health Organization (2018), depression affects more than 300 million people worldwide. Many of those also suffer from anxiety. This does not only have a negative impact in the health of those suffering from them, but also on their life quality. Furthermore, they also affect the economic well-being of entire regions due to a considerable decrease in productivity at work, and both welfare and health expenses [1]. Anxiety is characterized by an uneasy feeling about the future, including fear and uncertainty [2]. On the other hand, depression is a serious mood disorder with severe symptoms such as sad and anxious mood, pessimism, irritability, fatigue, alterations in sleeping patterns, and suicidal thoughts. Current research states that both disorders are triggered by the interaction of psychological, environmental, genetic, and biological factors. There exist many therapeutic options to treat these disorders. However, these options often take a long time to work, cause mood swings, alterations in sleeping patterns, dependence and addiction, and health affections in other parts of the body [3,4,5].

It is common to find both intestinal and mental disorders coexisting in the same individual. This suggests a strong connection between the central nervous system and the gastrointestinal tract [6]. By analyzing the complex communication system that exists between the gut and the brain, it was found that the relationship between these two organs goes further than just the maintenance of homeostasis. This association is referred to the gut-brain axis. Its role is to integrate intestinal functions and to link both the cognitive and the emotional cores of the brain with peripheral gut functions like the enteric reflex, intestine permeability, immune system stimulation, and enter-endocrine signaling. It is important to note that this relationship is bidirectional and that reciprocal brain-gut communication exists [7]. However, the understanding of this complex gut-brain interaction would be incomplete without considering the role that gut microbiota exerts.

There is evidence that suggests that the enteric microbiome plays a key role in the gut-brain axis communication. In fact, these microorganisms in the gut interact so closely with the host that they form a vital relationship that even controls homeostasis. Although each person has their own specific microbiota, a certain equilibrium is responsible for many essential functions. That is why when this balance is altered, some conditions that affect the gut-brain-endocrine relationship can arise, and eventually result in disease [8]. This gives rise to a new concept: The gut-brain-microbiota axis, which consists of a bilateral communication system that enables gut microbes to interact with the brain, and the latter with the gut. The mechanisms underlying this interaction pathway have not been completely elucidated, but strong evidence shows the involvement of neural, endocrine, immune, and metabolic systems [9].

The gut microbiota consists of more than 1 × 10^14^ microbe cells found in the human gastrointestinal tract, strongly reflecting evolutionary selection pressure at both host and microbial levels. These microorganisms play a determinant role in human health through the interaction between each other and with the host itself. In fact, this interaction could result in changes in both human physiological behavior and pathogenesis, including the correct functioning of the gastrointestinal tract and the central nervous system [10]. Intestinal bacteria imbalance has been observed in diseases outside the digestive system. The latter have the potential to affect the central nervous system, as well as many cognitive functions. There is evidence that suggests that enteric infections can cause both depression and anxiety. In fact, it has been found that gastrointestinal diseases, such as irritable bowel syndrome, are often accompanied by depression and/or anxiety disorders [11]. Moreover, it has been found that the gut bacteria influence behavior and that both depression and anxiety symptoms are directly associated with alterations in the microbiota. This promises a novel approach to preventing and treating mental disorders [12].

Psychobiotics are defined as probiotics that confer mental health benefits to the host when consumed in a particular quantity through the interaction with commensal gut bacteria [13]. As it has been stated, depression and anxiety are disorders with high prevalence worldwide. Although there exists a wide array of therapeutic options to treat them, undesirable secondary effects accompany most of them. The two main research questions that this work intends to answer include: What recent evidence is there in the literature regarding the psychobiotic effect that specific strains or formulations possess when ingested in a suspension or through a fermented product as well as the possible underlying mechanisms regarding such effect, and whether there exists a consensus or systemic methodology to assess the psychobiotic effect of food products or strains. In this regard, a systematic research was carried out through two different scientific databases using gut, brain, microbiota, axis, and psychobiotic as search criteria. In the first database (Scopus), 718 articles were found under those terms, while 72 were found using PubMed. In both cases, recent articles were prioritized, as well as original articles over review articles. Therefore, this review explores the potential of psychobiotics as a possible treatment against mental disorders, and explores their current applications in food. Moreover, studies evaluating psychobiotic potential of bacterial strains are increasing in number, being a trending topic in the context of the usage of probiotics to enhance mental health. Thus, it is interesting to carry out a systemic analysis that allows us to correlate probiotic strains with a specific mechanism of action and its effect on specific mental disorders. Finally, based on the literature consulted, a broad methodology is proposed to evaluate a potential psychobiotic microorganism, encompassing in vitro and in vivo experiments.

## 2. Action Mechanisms

**Figure 1:**
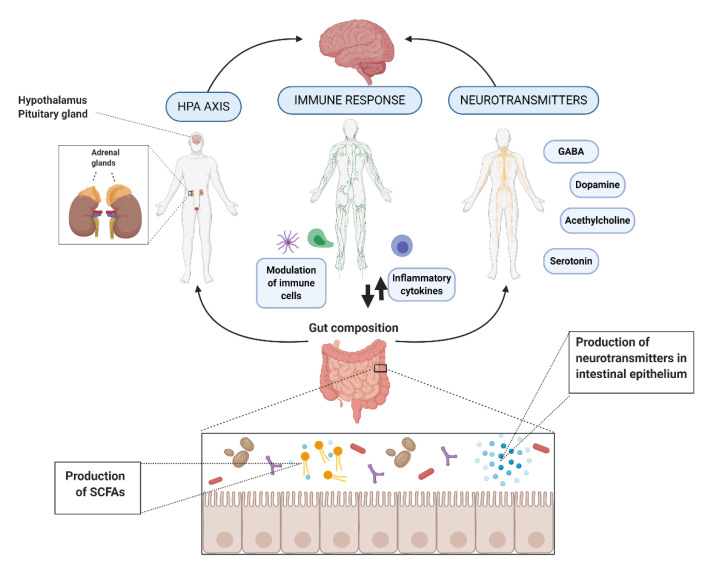
Action mechanisms by which the gut microbiota exert the potential psychobiotic effect.

The action mechanisms by which bacteria exert their psychobiotic potential has not been completely elucidated. However, it has been found that these bacteria provide their benefits through the enteric nervous system or the immune system’s stimulation. Furthermore, they affect the psychophysiological markers of depression and anxiety. This can happen in three different ways, first, by affecting the hypothalamic-pituitary-adrenal (HPA) axis stress response and reducing systemic inflammation; second, by a direct effect on the immune system; third, by the secretion of molecules such as neurotransmitters, proteins, and short fatty acids chains [14]. A graphic summary of the action mechanisms is shown in Figure 1.

### 2.1. Hypothalamic-Pituitary-Adrenal (HPA) Axis

The HPA axis is the primary neuroendocrine response system to physiological and physical stress in the human body. It includes the hypothalamus, pituitary gland, and adrenal cortex, as well as some regulatory inputs and secreted factors and hormones, such as cortisol in humans and corticosterone in rats [15]. Cortisol possesses immunosuppressant properties. Under chronic stress, cortisol is over-produced. However, it cannot exert its anti-inflammatory effects. As a result, cortisol’s negative feedback on the HPA axis is obstructed, resulting in hypercortisolemia [16]. This excess of glucocorticoids inhibits immunological activity. It also increases threat sensitivity and negative mood, impairs memory, and other cognitive functions [14]. Recent evidence suggests a strong bidirectional communication pathway between this neuroendocrine system and the gut microbiota. The gut colonization by microbes in early life has been identified to influence several aspects of both the brain and behavior, including the stress response. It has been found that HPA behavior is able to affect the gut microbiota composition and increase gastrointestinal permeability. It is possible to assume that any changes in the intestinal permeability and immune system may also play an important role in neuroendocrine malfunctions [17].

Gut microbiota imbalance can lead to the activation of the HPA axis. Thus, restoring this balance shows promising effects in downregulating the HPA axis. For instance, Ait-Belgnaoui et al. (2014) carried out an experiment where the effect of a probiotic formulation containing *Lactobacillus helveticus* R0052 and *Bifidobacterium longum* R0175 on the HPA axis response to chronic stress was assessed. They found that this probiotic supplementation significantly managed to attenuate the HPA axis response to stress [18]. Similar results were also observed by Andersson et al. (2016), but assessing the effect of *Lactobacillus plantarum* 299v on the HPA axis response to stress in humans. A randomized double-blind, placebo-controlled study measured the effect of this bacterial strain on the salivary cortisol levels in young adults under school-related chronic stress. This showed that the placebo group had higher salivary cortisol levels, proving that balanced gut microbiota can ameliorate the effects of chronic stress exposure through the modulation of the HPA axis [19].

### 2.2. Immune Response and Inflammation

Gut microbial dysbiosis is often connected to aberrant immune responses that include the overproduction of inflammatory cytokines. Microorganisms in the gut help to calibrate the innate and adaptive responses mainly by the production of small molecules that modulate host-microbiota interactions. Although the epithelial barrier prevents the escape of microorganisms from the gut, the metabolites they produce can pass through this barrier and enter and accumulate in the host’s circulatory system, where they are able to stimulate cells from the immune system [20]. Moreover, the gut microbiota has a strong influence on the population, migration, and function of various immune cells. Some publications have demonstrated the way the gut microorganisms modulate both innate and adaptive immune responses at the mucosal surface during infection, inflammation, and autoimmunity; specifically, how the communication pathways between the intestinal epithelium, the intestinal immune system, and gut microbes manage to regulate systemic immunity [21]. Microglia are the primary innate immune effector cells of the central nervous system [22]. It was recently found that the intestinal microbiota plays a crucial role in microglia maturation, morphology, and immunological function. This is because short-chain fatty acids (SCFA) are able to interact and regulate the correct functioning and development of the microglia [23].

Recent evidence suggests that higher levels of inflammation increase the risk of developing psychological disorders. In fact, higher levels of inflammatory cytokines, such as interleukin-6 (IL-6), IL-1β, and tumor necrosis factor-α (TNF-α), have been observed in depressed patients. Additionally, it has been observed that there is a positive association between microbiota composition and serum levels of interleukin-1α and interferon-γ, which are found to be positively correlated with depressive behavior [10]. In a recent study, Leclercq et al. (2017) investigated the long-term effect that antibiotics administration has on rodents at an early-life stage on both brain neurochemistry and behavior. They concluded that the antibiotic supplementation has a lasting effect on the gut microbiota composition and, consequently, increases the expression of cytokines in the frontal cortex, modifies the function of the blood-brain barrier, and even alters behavior. Furthermore, these mice exhibited impaired anxiety and social behaviors as well as increased levels of aggression. It is important to note that in the same study, one experimental group was also supplemented with *Lactobacillus rhamnosus* JB-1, which was shown to prevent some of the previously mentioned alterations [24].

### 2.3. Neurohormones and Neurotransmitters

The microbiome can produce a range of neuroactive compounds. Some neurochemicals that have been isolated from gut bacteria are gamma-aminobutyric acid (GABA), noradrenaline, serotonin, dopamine, and acetylcholine, which may affect the brain activity directly. Other bacterial metabolites with neuroactive functions include long and short-chain fatty acids. Hence, the capacity of some bacteria within the human gastrointestinal tract to produce and deliver neurotransmitters and neuromodulators has been suggested as a novel treatment for neuropsychiatric diseases [25].

#### 2.3.1. Serotonin

Serotonin (5-HT; 5-hydroxytryptamine) is a neurotransmitter involved in regulating behavioral and biological functions in the body like the mood. Additionally, it plays a role in both psychological processes in the central nervous system (CNS) and peripheral tissues such as the bone and gut [26]. 5-HT is primarily found in the intestinal mucosa, 90%–95% of serotonin is contained in two primary reservoirs: In the intestinal epithelium, where it is produced by enterochromaffin cells (ECs), and in neurons of the enteric nervous system. The reason for this location is largely unknown, although it may play a role in normal gut functions, including intestinal motility, absorption, and transit [27]. Microbiota promotes 5-HT biosynthesis from ECs, and it has been observed that microbiota-dependent effects on gut 5-HT modulated GI motility and platelet function [28,29].

Around 90% of the essential amino acid tryptophan is metabolized along the kynurenine pathway. Changes in the supply and availability of tryptophan have many implications in the enteric nervous system, CNS, and brain-gut axis signaling [30]. Clarke et al. (2012) reported that concentrations of tryptophan increased in the plasma of male germ-free animals, suggesting a humoral route through which the microbiota can influence CNS serotonergic neurotransmission [31].

#### 2.3.2. Dopamine and Epinephrine

Catecholamine (CA) neurotransmitters (dopamine (DA), norepinephrine (NE), and epinephrine (EP)) are biogenic amines derived from the amino acid tyrosine. They play vital roles in motor control, learning, memory formation, and in stress response. They also have profound effects on the cardiovascular system, by regulating the carbohydrates and fats metabolisms in the body [32,33]. NE and DA stand out as prefrontal cortex-dependent function regulators, such as attention, decision making, and inhibitory control. Prefrontal cortex dysfunction has been recognized as a central feature of many psychiatric disorders, among them schizophrenia, attention deficit hyperactivity disorder (ADHD), post-traumatic stress disorder (PTSD), and drug addiction [34].

Gut microbiota plays a critical role in the generation of free CA in the gut lumen. Biologically active DA and NE were identified in the gut lumen of specific-pathogen-free mice (SPF-M); also, the association with either a mixture of *Clostridium* species or fecal microbiota from SPF mice resulted in a drastic elevation of free DA and NE [35]. Moreover, gut microbes have been reported as an essential source of luminal NE; bacteria residing in the gut lumen retain uptake systems that act as a net sink for biogenic amines and neuroactive substances [36].

#### 2.3.3. Gamma-Aminobutyric Acid (GABA) and Glutamate

GABA and glutamate are the primary neurotransmitters of the mammalian CNS, whose role is to control excitatory and inhibitory neurotransmission. Coordination between these two neurotransmitters is essential for the normal functioning of complex brain processes such as neuronal excitability, synaptic plasticity, and cognitive functions such as learning and memory [37].

Multiple studies have reported GABA-producing microorganisms, mostly lactic acid bacteria (LAB). For instance, *Lactobacillus paracasei* PF6, *Lactobacillus delbrueckii* subsp. *bulgaricus* PR1, *Lactococcus lactis* PU1, and *Lactobacillus brevis* PM17 were isolated from Italian cheeses and shown to produce high GABA concentrations during the fermentation of reconstituted skimmed milk [38]. Recently, Valenzuela et al. (2019) examined the ability to produce GABA in LAB strains isolated from traditional dairy products made from raw milk, reporting more than 1 mM of GABA produced by strains of *Lactococcus lactis* subsp. *lactis* and *Streptococcus thermophilus* [39]. Moreover, it was found that GABA receptors are present in gut microbiome and that glutamic acid decarboxylase genes are distributed among *Lactobacillus plantarum, Lactobacillus brevis, Bifidobacterium adolescentis, Bifidobacterium angulatum, Bifidobacterium dentium,* and other gut-derived bacterial species, demonstrating its ability to produce GABA [40]. Furthermore, it has been shown that *Lactobacillus brevis* CRL 2013 is able to grow and produce high amounts of GABA in hexoses-supplemented MRS broth, such as glucose or fructose (Cataldo et al., 2020). Lastly, a transcriptome analysis of human stool samples from healthy individuals showed that GABA-producing pathways are actively expressed by *Bacteroides* [41].

#### 2.3.4. Acetylcholine

Acetylcholine (ACh) has a role as the primary excitatory neurotransmitter in the periphery. It appears to act as a neuromodulator in the brain: Influences synaptic plasticity, reinforces neuronal loops and cortical dynamics during learning, changes neuronal excitability, and can also alter the firing of neurons on a rapid time scale in response to changing environmental conditions [42]. Moreover, ACh and the enzymes participating in the acetylcholine synthesis have been well identified as components of bacteria. Its production was discovered for the first time in a strain of *Lactobacillus plantarum* [43].

### 2.4. Intermediate Substances and Metabolites

#### 2.4.1. Vagus Nerve

The vagus nerve (VN) is the longest cranial nerve in the body, containing sensory fibers that deliver information to the brain from the heart, lungs, pancreas, liver, stomach, and intestines. VN consists of sensory (afferent) and motor (efferent) neurons. Vagal afferent terminals are positioned beneath the gut epithelium to receive, directly or indirectly, signals produced by the gut microbiota, to affect host behaviors. These behaviors include lethargy, depression, anxiety, and loss of appetite, among others [44].

The connection with the enteric nervous system, which governs the gastrointestinal tract function, widens the scope of signals that can be transmitted through the VN by microorganisms. Vagal afferent fibers sense microbiota signals indirectly, through the diffusion of bacterial compounds or metabolites or other cells located in the epithelium that relay luminal signals. For instance, it has been reported that specific bacterial strains utilize vagus nerve signaling to communicate with the brain and to alter behavior. In addition, the vagal activity provides a protective function to the intestinal epithelial barrier; low vagal activity makes intestinal epithelium more permeable, thus promoting systemic inflammation and chronic disease [45,46].

#### 2.4.2. Brain-Derived Neurotrophic Factor (BDNF)

BDNF is a neurotrophin structurally related to nerve growth factor, neurotrophin-3, and neurotrophin-4, which regulate the viability and functional integrity of specific neuronal populations. BDNF involves functions within the CNS, including neuronal survival and differentiation. Changes in BDNF levels may contribute to the dysfunction of synaptic transmission and plasticity [47]. Gut microbiota influences the expression of BDNF in brain regions crucially involved in the development of correct behavioral patterns. Several studies suggest that the gastrointestinal microbiota may influence behavior by modulating BDNF production in the CNS [48].

#### 2.4.3. Short-Chain Fatty Acids (SCFAs)

SCFAs are saturated aliphatic organic acids that consist of one to six carbons; acetate, propionate, and butyrate are the most abundant, and are present in the colon and stool. SCFA are produced by gut bacteria through saccharolytic fermentation of carbohydrates that escape digestion and absorption in the small intestine. The different types and amounts of nondigestible carbohydrates that reach the cecum and large intestine depend on the daily intake and type of food, mainly fiber. The amount and type of fiber consumed have significant effects on the intestinal microbiota composition and, therefore, the type and amount of SCFAs produced [49]. Regarding its role in the organism, SCFAs act as metabolic substrates regulating host cellular metabolism, appear to play an important role in regulating the integrity of the epithelial barrier, regulating the immune system and inflammatory response, and eliciting effects on lipid metabolism and adipose tissue at several levels [50]. Moreover, SCFAs might directly influence neural function by reinforcing blood-brain barrier integrity, modulating neurotransmission, influencing levels of neurotrophic factors, and promoting memory consolidation. Increased evidence suggests a potential key role of SCFAs in gut-brain axis signaling [51].

## 3. Evaluation Methods

**Figure 2:**
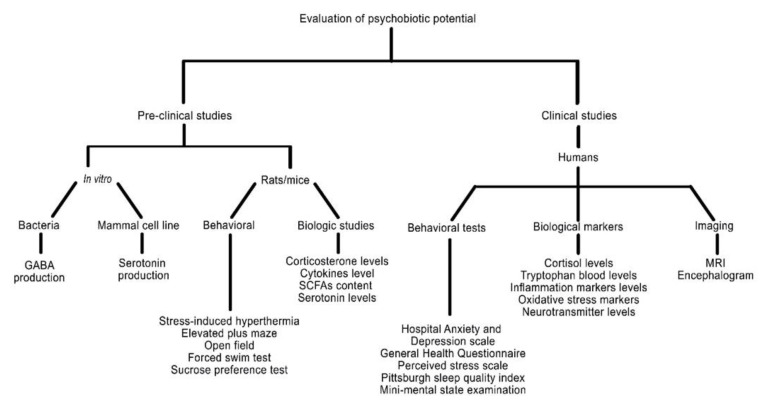
Diagram of preclinical and clinical tests used to evaluate psychobiotic potential. GABA, gamma-aminobutyric acid; SCFAs, Short Chain Fatty Acids; MRI, magnetic resonance imaging.

After an extensive revision in the literature, we consider that no synthesis of experiments has been reported to assess psychobiotic potential. Therefore, we reviewed a series of tests that have been applied more commonly to evaluate the potential of a strain, formulation, or fermented product to modulate the gut microbiota composition, human or animal behavior, and mood disorders such as anxiety or stress. These tests are divided into preclinical as well as clinical trials in humans. This information is outlined in Figure 2 and described in detail in the Supplementary Table S1, where a table is included in which the most common tests are presented according to the type of study, experimental subject, and type of test, as well as the references where each test has been used.

The in vitro preclinical studies in bacteria and mammal cell lines monitor and measure the production of neurotransmitters among other bioactive compounds. Moreover, in the preclinical section, several behavioral and biological studies were used to assess the psychobiotic effect of strains, formulations, or fermented products in rats and mice. Some of the behavioral studies employed the most include the elevated plus maze, the open field test, and the forced swim test; focusing on analyzing animal conduct in response to stress. In addition to the behavioral studies, certain biologic compounds related to psychobiotic potential measurements were also conducted in animal models, such as the quantification of corticosterone and cytokines levels, and the abundance of SCFAs and serotonin in different tissues.

Furthermore, clinical studies are also reported in the literature to study the psychobiotic potential of microorganisms in different presentations. As well as with the animal studies, they can be divided into behavioral tests and biological markers evaluation with the imaging section addition. The behavioral tests aimed to measure the psychosomatic state of the patient, as well as how the latter had an impact on their daily life activities. Some of these tests include the Hospital Anxiety and Depression Scale, the Perceived Stress Scale, and even the Pittsburgh Sleep Quality Index. In order to further understand the psychobiotic potential effect, the cortisol, tryptophan, and neurotransmitter levels were observed in the patients as well as some inflammation and oxidative stress markers. Clinical studies also include imaging studies such as magnetic resonance imaging (MRI) and encephalograms to patients, in contrast to pre-clinical studies.

## 4. Action Mechanisms Regarding the Psychobiotic Potential of Specific Strains and Formulations

There are several studies in the literature regarding the psychobiotic potential of several bacterial strains by themselves as well as some formulations (cocultures). This section addresses the psychobiotic effect of both strains and formulations by emphasizing the possible underlying mechanism attributed to the psychobiotic potential of each specific strain or formulation, the methodology that was followed, and the most remarkable results and conclusions found by the authors. A summary of this information is shown in Table 1. However, it is important to note that the authors did not follow a systematic methodology whatsoever to assess the psychobiotic potential of a strain or formulation, and that each of them choose the tests or assays that they find more suitable for them, which results in a very variable, non-homogenous battery of experiments. In this sense, this leads to a wide variety of results that are difficult to compare and contrast within each other or even to classify them according to specific criteria. 

### 4.1. Bacteria Strains with Psychobiotic Potential According to Its Possible Action Mechanism

In order to determine the effect of daily supplementation of *Lactobacillus casei* Shirota on stress and anxiety in athletes, 20 male football players took part in a study where they were given daily a probiotic stick containing 1 × 10^9^ CFU of the *Lactobacillus casei* Shirota strain, for 8 weeks. Anxiety and perceived stress were measured at baseline, week 4, and week 8 using the competitive state anxiety inventory and the perceived stress scale. It was concluded that the daily psychobiotic supplementation significantly reduced the cognitive state anxiety scores, somatic state anxiety, and perceived stress scores [56]. However, during this experiment, no biological markers were measured, so the authors were not able to identify the mechanism by which *Lactobacillus casei* Shirota managed to reduce anxiety in the subjects.

In a similar study, Miyaoka et al. (2018) carried out an experiment in order to evaluate the effects of *Clostridium butyricum* MIYAIRI 588 as adjunctive therapy for treatment-resistant major depressive disorder. Patients were randomized to adjuvant treatment with psychobiotic supplementation (60 mg daily) or control. To assess any significant changes, the 17-item Hamilton Depression Rating Scale was used as well as the Beck Depression Inventory (BDI) and the Beck Anxiety Inventory (BAI) scale scores. The results showed that psychobiotic supplementation, in combination with antidepressants, provided a significant improvement in depression. 70% of patients responded favorably to treatment with a remission rate of 35%. A more than a 50% reduction in the 17-item Hamilton Depression Rating Scale scores, BDI scores, and BAI scores at the end of the 8-week trial, regardless of the type of antidepressant drug used, was observed. They were able to conclude that adjunctive *Clostridium butyricum* MIYAIRI 588 exhibited a greater magnitude of treatment effectiveness even in those patients with more severe depression disorders who had failed to achieve an adequate response to previous antidepressant therapy. Even though they did not focus their study on measuring changes in biological factors to elucidate any possible mechanism that could explain their findings, the authors were able to conclude through a literary research that *Clostridium butyricum* has powerful neuroprotective and anti-inflammatory effects [55].

Nishida et al. (2017) investigated the psychobiotic potential of *Lactobacillus gasseri* CP2305 to ameliorate chronic stress-associated symptoms in medical students. However, it is important to note that the bacteria strain had been inactivated before the consumption by the experimental subject, meaning it was no longer considered a probiotic but a parabiotic. Each student drank daily, either a placebo or a beverage containing 1 × 10^10^ bacterial cells for 12 weeks. All subjects were asked to keep track of their physical and mental health using questionnaires such as the 28 item General Health Questionnaire, the Hospital Anxiety and Depression Scale, the Spielberger State-Trait Anxiety Inventory, and the Pittsburgh Sleep Quality Index (PSQI). Moreover, the students were also subjected to biological stress response measurements like basal salivary cortisol levels, sleep electroencephalogram, autonomic nervous activities, and stress-responsive miRNAs in circulating leukocytes. Some of the most relevant results include a desirable change in the PSQI for the parabiotic group and a reduction on the State-Trait Anxiety Inventory. Regarding the salivary cortisol levels, the parabiotic ingestion significantly suppressed the cortisol levels’ escalation compared to the placebo group. Through a careful examination of the results, the authors concluded that the stress-relief effect is related to anti-inflammatory mechanisms associated with changes in the microbiota composition induced by the parabiotic intake [58].

Bravo et al. (2011) carried out a study to assess whether *Lactobacillus rhmanosus* JB-1 could mediate direct effects on the GABAergic system and to evaluate any behaviors relevant to GABAergic neurotransmission and stress response. Mice were orally gavaged with *Lactobacillus rhamnosus* JB-1, containing 1 × 10^9^ CFU for 28 continuous days. Then, a battery of behavioral tests relevant to anxiety and depression such as the stress-induced hyperthermia and elevated plus maze tests were performed. The biological markers that were analyzed in the present study were corticosterone levels and GABA receptor expression. The probiotic administration reduced the content of corticosterone and restricted behaviors associated with depression and anxiety. This strain’s neurochemical and behavioral effects were absent in mice after vagotomy, indicating that the VN is a critical element of communication between the intestinal microbiota and the central nervous system [67]. In a similar study, Kantak, Bobrow, and Nyby (2014) assessed the psychobiotic effect of the *Lactobacillus rhamnosus* GG. To achieve this, male BALB/cJ mice were supplemented with this strain at a density of 1 × 10^9^ CFU/day for 2 weeks before an injection of the serotonin antagonist RU 24969 known to induce obsessive-compulsive disorder (OCD)-like behaviors in this specific strain of mice. The *Lactobacillus rhamnosus* GG treatment group was compared against a placebo group and a fluoxetine group. The authors were able to conclude that *Lactobacillus rhamnosus* GG had normalizing effects on hyperlocomotion, stereotypic turning, thigmotaxis, and perseverative marble burying. Additionally, *Lactobacillus rhamnosus* supplementation seemed to show therapeutically comparable results with fluoxetine, a standard treatment for obsessive-compulsive disorder in humans. Even though no biological marker measurements were carried out whatsoever, the authors concluded that the psychobiotic effect was mainly due to changes in brain serotonin signaling, similar to the effect observed in fluoxetine intake [68].

There have been a few studies where more than one possible action mechanism is identified. For example, in a preclinical study, Luo et al. (2014) tested the efficacy of probiotic *Lactobacillus helveticus* NS8 in preventing cognitive decline and anxiety-like behavior in rats with hyperammonemia-induced neuroinflammation. The rats were given a bacterial suspension in sterile water containing 1 × 10^9^ CFU of *Lactobacillus helveticus* NS8 per mL for 3 weeks. There was no restriction in the amount of the suspension, but the daily dosage of the strain was measured by the amount of water consumed daily. The anxiety behavior and cognitive function were tested using the elevated plus maze and Morris water maze. Neuroinflammatory markers were measured as well, including prostaglandin E2, inducible nitric oxide synthase, and interleukin-1 beta (IL-1β). Finally, the metabolic activity of brain serotonin and kynurenine pathway in plasma were also assessed. After analyzing the results, the authors concluded that probiotic treatment of rats with *Lactobacillus helveticus* NS8 improved cognitive decline and anxiety-like behavior. As for the biological markers, bacterial supplementation significantly reduced the levels of prostaglandin E2 in the cerebellum and the levels of IL-1β in the cerebellum, hippocampus, and prefrontal cortex [64]. Another way to induce anxiety-like behavior in rats is shown in a study performed by Desbonnet et al. (2010), where the potential antidepressant effect of *Bifidobacterium infantis* 35,624 was assessed in the rat maternal separation model, which has proven to be useful in the study of stress-related gastrointestinal affections and mood disorders. Maternally separated adult rat offspring were treated with either a *Bifidobacterium infantis* 35624 oral suspension (1 × 10^10^ live bacterial cells in 100 mL of the rats drinking water) or citalopram daily. Afterward, they were subjected to the forced swimming test. Furthermore, cytokine concentrations, monoamine levels in the brain, and central and peripheral HPA axis measures were also analyzed. It was observed that maternal separation reduced swim behavior and increased immobility during the force swimming test, decreased noradrenaline levels in the brain, and promoted the release of peripheral IL-6 while increasing the contents of the amygdala corticotrophin-releasing factor mRNA. However, when the probiotic supplementation was done accordingly, the immune response was normalized, the behavioral deficits reversed, and basal noradrenaline levels were reestablished in the brain [62].

Another example where multiple action mechanisms have been identified is the study carried out by Liu et al. (2016), where the psychobiotic potential and psychotropic effects of *Lactobacillus plantarum* PS128 was investigated. To do so, naïve adult mice and mice subjected to early-life stress were administered with 1 × 10^9^ CFU resuspended in saline solution by gavage for 4 weeks. Afterward, the mice underwent a battery of behavioral tests. The tests were given in sequence from the least stressful to the most stressful in the following order: Sucrose preference test, open field test, elevated plus maze, and forced swimming test. Biological markers, such as serum corticosterone, serum cytokine levels, monoamines, and metabolites were measured. The behavioral tests showed that the supplementation of *Lactobacillus plantarum* of PS128 increased the locomotor activities in both early-life stressed and naïve adult mice in the open field test. Regarding the elevated plus maze, *Lactobacillus plantarum* PS128 significantly reduced the anxiety-like behaviors in naïve adult mice, but not in the early-life stressed mice. On the other hand, depressive behaviors were reduced in early life stressed mice, but not in naïve mice. *Lactobacillus plantarum* administration also reduced serum corticosterone’s elevation under both basal and stressed states in early-life stressed mice, but did not affect naive mice. Furthermore, psychobiotic supplementation significantly reduced inflammatory cytokine levels and increased anti-inflammatory cytokine levels in the serum of early-life stressed mice. Finally, the dopamine level in the prefrontal cortex was significantly increased in both treatment groups, while serotonin level was increased only in the naïve adult mice [66].

In a recent study, Tian et al. (2020) researched upon the psychobiotic potential of *Bifidobacterium breve* CCFM1025. Chronically stressed C57BL/6J male mice were fed with 0.1 mL/10 g body weight of *Bifidobacterium breve* CCFM1025 suspension at a concentration of 1 × 10^9^ CFU/mL daily for 5 weeks prior to a battery of behavioral tests. Moreover, brain neurological alterations, serum corticosterone, cytokines levels, fecal microbial composition, and SCFA content were measured as well. At last, the effect of SCFAs on 5-hydroxytryptophan (5-HTP) biosynthesis was investigated in an in vitro model of enterochromaffin cells. Some of the most relevant results included that the *Bifidobacterium breve* CCFM1025 treatment significantly decreased anxious and depressive behaviors. Additionally, they observed that the psychobiotic supplementation mitigated the inflammation induced by HPA axis hyperreactivity, which reflected on a significant reduction of the expression of the glucocorticoid receptor, hippocampal IL-6 levels, and circulating TNF-α. Additionally, an increase in the regulation of BDNF was identified. Finally, the authors were also able to conclude that the psychobiotic supplementation managed to restore the equilibrium on the gut microbiota, specifically by the ratio of Actinobacteria to Proteobacteria [61]. We can observe that Hao, Wang, Guo, and Liu (2019) performed a similar preclinical study whose main objective was to test if *Faecalibacterium prausnitzii* ATCC 27766 had psychobiotic potential in ameliorating both anxiety and depression symptoms, and whether it could reverse the impact of chronic unpredictable mild stress in rats. To fulfill their objective, 60 male rats were divided into 3 groups: Untreated rats, rats exposed to stress, and rats exposed to stress and treated with a *Faecalibacterium. prausnitzii* (ATCC 27766) suspension. Rats were fed with 200 µL of a phospate-buffered saline (PBS) suspension containing 1 × 10^9^ CFU daily for 4 weeks by oral gavage. The depression and anxiety behavioral tests included the elevated-plus maze, the open field test, and the forced swim test. Some physiological stress-related factors were assessed, such as growth status, SCFA production, plasma cytokine levels, endocrinology, and even bone mineral density. After analyzing the results, the authors concluded that the administration of *Faecalibacterium prausnitzii* has both preventive and therapeutic effects on depression and anxiety symptoms in rats that were exposed to chronic unpredictable mild stress. This was because the group that received the psychobiotic treatment had higher levels of SCFAs in the cecum and higher levels of cytokine interleukin-10 in the plasma. Moreover, *Faecalibacterium prausnitzii* supplementation prevented the stress-related effects of corticosterone, C-reaction protein, and cytokine IL-6 release [63].

Savignac, Kiely, Dinan, and Cryan (2014) investigated the psychobiotic potential of *Bifidobacterium breve* 1205, and *Bifidobacterium longum* 1714 to alter the behavior of BALB/c mice. To achieve this, the innately anxious mice were orally fed with a bacterial suspension of *Bifidobacterium breve* 1205 at a concentration of 1 × 10^9^ CFU/mL, *Bifidobacterium longum* 1714 at a concentration of 1 × 10^9^ CFU/mL, escitalopram (an antidepressant), or sterile PBS, for 3 weeks. A wide array of behavioral tests was used to measure the differences in anxiety symptoms among the groups, such as stress-induced hyperthermia, defensive marble burying, elevated plus maze, open field, tail suspension test, and forced swim test. Some stress-related physiological parameters were also measured, which include corticosterone plasma levels and the weight of certain organs like thymus, heart, adrenals, and spleen. The results showed that *Bifidobacterium breve* 1205 improved behavior relevant to stress in innately anxious mice. Specifically, it managed to induce an anxiolytic effect in the elevated plus maze and reduced bodyweight gain, suggesting a role in general anxiety and metabolism. Spleen weight was altered by both escitalopram and *Bifidobacterium breve* 1205, while the other parameters remained the same. This suggests the possible involvement of the brain-gut axis and the potential systems involved behind the behavioral changes observed. On the other hand, *Bifidobacterium longum* 1714 decreased stress-induced hyperthermia and antidepressant-like behavior in the tail suspension test, suggesting a positive role in sensitivity to acute stress and depression [60]. In regard to the psychobiotic effect of *Bifidobacterium longum* 1714, recent preclinical studies have identified the *Bifidobacterium longum* 1714 strain as a potential psychobiotic. However, in order to observe whether that this claim could be translated into human volunteers, Allen et al. (2016) studied if the regular consumption of *Bifidobacterium longum* 1714 strain had any effect on the stress response, cognition, and brain activity of human volunteers. To exclude the effects of individual differences across variables, a repeated measures design was used. Each participant took a placebo for four weeks, followed by the probiotic supplementation for another four weeks at a 1 × 10^9^ CFU concentration. To assess any significant changes, the volunteers completed some cognitive tests, resting encephalography, and were subjected to a socially evaluated cold pressor test at baseline, post-placebo, and post-psychobiotic. It was observed that after the psychobiotic supplementation, the increases in cortisol output and subjective anxiety in response to the socially evaluated cold pressor test were attenuated. Furthermore, daily reported stress was reduced by *Bifidobacterium longum* 1714 consumption. Subtle improvements in hippocampus-dependent visuospatial memory performance were also found, as well as enhanced frontal midline electroencephalographic mobility after the psychobiotic consumption [53].

In a more complex experiment, Takada et al. (2016) aimed to examine the effects of *Lactobacillus casei* strain Shirota on gut–brain interactions under stressful conditions in both human and animal models. For the animal study, adult male F344 rats were given 3 × 10^9^ CFU in their feed daily or a placebo for 2 weeks. Then, the rats were submitted to the water avoidance stress test. To assess any psychobiotic effect, the plasma corticosterone levels were analyzed, as well as some immunohistochemical assays in the nervous system and electrophysiological recording of gastric vagal afferent activity. They observed that *Lactobacillus casei* supplementation significantly suppressed increases in corticosterone levels when exposed to the water avoidance stress test. As for the gastric vagal afferent activity, it was improved in the group that received the presumed psychobiotic strain when compared to the placebo one. For the clinical part of the study, healthy medical students preparing to take an important exam were given either milk fermented with *Lactobacillus casei* Shirota (1 × 10^9^ CFU/mL) or non-fermented placebo milk for 8 weeks in a double-blind, placebo-controlled, parallel-group trials study. During the experiments, the subjects were asked to fill daily and weekly questionnaires regarding any physical discomfort. Additionally, saliva was collected at baseline, at 6 weeks into the intervention, on the day before the exam, and immediately after the exam on the same day to measure cortisol levels. The authors observed that the cortisol changed from baseline was significantly lower in the psychobiotic group than the placebo group the day before the exam. Regarding the incidence of physical symptoms, in the *Lactobacillus casei* Shirota-supplemented group, the rate of flu symptoms and the incidence rate of abdominal symptoms were significantly lower compared with those of the placebo group [59].

Depression and anxiety are sometimes accompanied by other diseases. For instance, a strong link between type 2 diabetes mellitus and psychiatric disorders like depression and anxiety has been identified, and the association of the gut–brain axis in the development of these disorders has been thoroughly elucidated. Morshedi et al., (2018) carried out a preclinical study to assess the beneficial psychological effects of *Lactobacillus plantarum* ATCC 8014 in diabetic rats. They evaluated not only the psychobiotic potential of *Lactobacillus plantarum* ATCC 8014, but also if this potential could be enhanced by adding a prebiotic, inulin, to the formulation. The rats were divided into 5 groups: Healthy control, *Lactobacillus plantarum,* inulin, *Lactobacillus plantarum* + inulin, and diabetic sham group. The ATCC 8014 groups received a bacterial suspension of 1 × 10^7^ CFU/mL through gastric gavage daily. In contrast, the inulin groups received 5% of their daily food weight in inulin dissolved in their drinking water. The rats underwent the elevated plus maze test and the forced swimming test to identify any behavioral changes. Some biochemical assays were performed, such as measuring the oxidative markers of the blood and amygdala and the concentrations of amygdala serotonin and brain-derived neurotrophic factor (BDNF) in the rats. It was found that the administration of these supplements in diabetic rats for 8 weeks could ameliorate oxidative stress status, insulin, and fasting blood sugar levels. In addition, rats that received both inulin and *Lactobacillus plantarum* ATCC 8014 showed an increase in the amygdala BDNF and serotonin levels, which resulted in improved depressive and anxiety-like behaviors. Furthermore, beneficial effects were observed on the elevated plus maze and forced swimming tests, where no change in the rats’ locomotor activity was observed whatsoever [65]. Another example of diseases correlated with depression or anxiety is irritable bowel syndrome (IBS). For that matter, Pinto-Sanchez et al. (2017) investigated the psychobiotic potential of *Bifidobacterium longum* NCC3001 on anxiety and depression in patients suffering from IBS. For that matter, 44 adults with IBS and mild to moderate anxiety and/or depression took part in a randomized, double-blind, placebo-controlled study. Patients were randomly assigned to groups and given daily the bacterial suspension at a concentration of 1 × 10^10^ CFU or placebo for 6 weeks. At weeks 0, 6, and 10, the patient levels of anxiety and depression were assessed, as well as IBS symptoms, quality of life, and somatization, through the use of questionnaires. Moreover, at weeks 0 and 6, stool, urine, and blood samples were collected, and functional magnetic resonance imaging (fMRI) test was performed. All of this to observe brain activation patterns, fecal microbiota, urine metabolome profiles, serum markers of inflammation, neurotransmitters, and neurotrophin levels. At week 6, more than 60% of the patients in the experimental group had a reduction in depression scores on the Hospital Anxiety and Depression scale. In comparison, only 30% of patients in the placebo group showed the same outcome. However, psychobiotic supplementation had no significant effect on anxiety or IBS symptoms in this case. On the other hand, the fMRI analysis showed that patients who took *Bifidobacterium longum* NCC3001 had reduced responses to negative emotional stimuli in multiple brain areas. At last, all the groups had similar fecal microbiota profiles, serum markers of inflammation, and levels of neurotrophins and neurotransmitters [54]. The effect of a psychobiotic strain on patients with IBS and a mental disorder was also assessed by Majeed, Nagabhushanam, Arumugam, Majeed, and Ali (2018). Only this time, they studied the effect of *Bifidobacterium coagulans* MTCC 5856 strain in patients with IBS and depressive disorder. To do so, forty patients diagnosed for both disorders were randomized to receive either 2 billion spores of *Bifidobacterium coagulans* MTCC 5856 or a placebo daily for 90 days. Any changes in the clinical symptoms were evaluated using a set of questionnaires, which include the Hamilton Rating Scale for Depression (HAM-D), Montgomery-Asberg Depression Rating Scale (MADRS), Center for Epidemiological Studies Depression Scale (CES-D), and Irritable bowel syndrome quality of life questionnaire (IBS-QOL). The results showed that psychobiotic supplementation reduced the symptoms for both disorders significantly compared to the placebo group. Furthermore, the authors also observed serum myeloperoxidase’s concentration, an inflammatory biomarker clinically relevant for depression and IBS, in the patients before and after the experiment. In fact, a significant reduction in the levels of this biomarker was observed in the experimental group, but not in the placebo one. Both results indicate that *Bifidobacterium coagulans* MTCC 5856 may be a novel alternative approach for managing major depression disorder in patients with IBS [52].

### 4.2. Bacterial Formulations with Psychobiotic Potential According to Its Possible Action Mechanism

Just like with the isolated strains, the underlying mechanism of action of bacterial formulations with psychobiotic potential is not fully understood yet. However, many authors have carried out measurements of biological markers in parallel to the behavior tests, in order to identify the possible reasons behind the psychotropic-like effect of the formulations.

For instance, Yunes et al. (2019) identified that both *Lactobacillus plantarum* 90sk and *Bifidobacterium adolescentis* 150 can produce relatively high amounts of GABA. Subsequently, the probiotic properties of the selected strains and their antidepressive effects in mice were evaluated. Mice were separated into four different groups: Fluoxetine, monosodium glutamate, distilled water, and probiotic formulation. The mice’s depressive-like behavior was assessed using the forced swimming test. It was observed that both the probiotic formulation and fluoxetine experimental groups spent significantly less time immobile than the control groups, which is strongly related to reduced depressive-like behavior. This is due to the fact that GABA directly modulates GABAergic signaling through GABA receptors found on enteric neurons [74].

In another study, Messaoudi et al. (2010) investigated the anxiolytic activity of a probiotic formulation containing *Lactobacillus helveticus* R0052 and *Bifidobacterium longum* R0175 in rats and its possible effects on anxiety, depression, stress, and coping mechanisms in healthy human volunteers. In the preclinical study, 36 male Wistar rats were distributed into three experimental groups. Placebo, positive control (diazepam), or a probiotic formulation containing 1 × 10^9^ CFU of *Lactobacillus helveticus* and *Bifidobacterium longum* were administered daily for 14 days. The results showed a significant difference in the stress/anxiety score among groups. Moreover, similar results were observed between rats fed with the probiotic formulation, and those with the positive control. For the clinical study a double-blind, controlled, and parallel experiment was conducted in 60 healthy Caucasian men and women. Three measurements were taken at week 0, week 2, and week 6. Some of the measured parameters include the Hopkins Symptom Checklist, Hospital Anxiety and Depression Scale, Perceived Stress Scale, the Coping Checklist, and measurement of urinary free cortisol. Some results include the higher percentage change on the global severity index in the probiotic formulation treated patients, and that the differences in the Hospital Anxiety and Depression Scale among groups before and after treatment were significant. It was also observed that the median urinary free cortisol level of probiotic formulation subjects decreased between week 2 and week 6. This decrease in urinary free cortisol, which is a HPA axis regulator, might be one of the reasons behind the psychobiotic effect of this formulation. However, the authors suggest further pre and clinical studies to better understand this mechanism [73].

As we can recall, there are several action mechanisms by which a bacterial formulation can exert a psychobiotic effect, such as modifications in the synthesis pathways of neurohormones and neurotransmitters. For example, Steenbergen, Sellaro, van Hemert, Bosch, and Colzato (2015) tested if a multispecies probiotic formulation containing *Bifidobacterium bifidum* W23, *Bifidobacterium lactis* W52, *Lactobacillus acidophilus* W37, *Lactobacillus brevis* W63, *Lactobacillus casei* W56, *Lactobacillus salivarius* W24, and *Lactococcus lactis* (W19 and W58) could reduce cognitive reactivity in non-depressed individuals. To achieve this, they conducted a triple-blind, placebo-controlled, randomized, pre- and post-intervention assessment design where 20 healthy participants received a 4-week probiotic food-supplement intervention with the probiotic formulation at a concentration of 2.5 × 10^9^ CFU/g, while 20 other participants received a placebo. In the pre- and post-intervention assessment, cognitive reactivity to sad mood was assessed through the Leiden index of depression sensitivity scale. In contrast with participants who received the placebo intervention, those who received the 4-week multispecies probiotics significantly reduced overall cognitive reactivity to sad mood. Even though the authors did not measure any biological markers to elucidate a possible action mechanism behind the psychobiotic effect, they did state that intestinal microbiota increase plasma tryptophan levels, facilitating serotonin turnover in the brain, which has been associated with cognitive reactivity to sad mood [70]. In a similar study, Kazemi, Noorbala, Azam, Eskandari, and Djafarian (2018) compared the effect of a probiotic formulation and a prebiotic on decreasing the Beck Depression Inventory (BDI) score in adult subjects with mild to moderate major depressive disorder (MDD) through a double-blind randomized controlled trial. They also measured the kynurenine/tryptophan ratio along with the tryptophan/branch chain amino acids ratio as secondary outcomes to investigate any changes in the metabolism of neurotransmitters. To achieve this, 110 depressed patients were randomly assigned to receive the probiotic formulation at a dosage of 10 billion CFU containing *Lactobacillus helveticus* R0052 and *Bifidobacterium longum* R0175, galactooligosaccharide, or a placebo for 8 weeks. Afterward, serum tryptophan and branch chain amino acids (BCAAs) were measured by high performance liquid chromatography (HPLC), while the kynurenine pathway metabolites were assessed with an ELISA kit. Probiotic formulation supplementation resulted in a significant decrease in the BDI score compared to the placebo and prebiotic supplementation groups. No significant differences among the groups in terms of serum kynurenine/tryptophan ratio and tryptophan/BCAAs ratio were identified. However, the kynurenine/tryptophan ratio decreased significantly in the probiotic group after adjusting for serum isoleucine. Moreover, the tryptophan/isoleucine ratio increased significantly in the probiotic group compared to the placebo group. Probiotics reduce the activity of enzymes that change tryptophan to kynurenine, which leads to higher levels of serotonin. Therefore, the decrease in the kynurenine/tryptophan ratio may be a mechanism for the observed effects on depression [69].

It is no surprise that the psychobiotic potential of a specific bacterial formulation can be explained through more than one possible mechanism. For instance, Tian, Wang, Zhao, Zhang, and Chen (2019) investigated the effect of probiotic treatment on depression using a murine model. Male adult C57BL/6J mice were exposed to chronic unpredictable mild stress for 5 weeks while taking a placebo, fluoxetine as a positive control, or the probiotic formulation containing *Bifidobacterium longum* subsp. *infantis* E41 and *Bifidobacterium breve* M2CF22M7 at a concentration of 1 × 10^9^ CFU suspended in sterile saline solution. The mice were subjected to a wide battery of behavioral tests like the forced swim test, tail suspension test, sucrose preference test, open field test, elevated plus maze, light/dark box test, and step-down test to investigate the cecal SCFAs levels and some neurobiological factors. The probiotic formulation supplementation significantly reduced depressive behaviors of mice in the forced swim test, sucrose preference test, and step-down test, through an increase in the levels of 5-hydroxytryptamine and BDNF concentration in the brain. This in turn is associated with regulation of the HPA axis, which was reflected by a decrease in serum corticosterone levels. Psychobiotic mechanisms can be so complex that even in the same study, the authors found a strong relationship between cecal SCFAs contents and depressive behavior [75].

Another example of formulation that exerts its psychobiotic potential through more than one mechanism is found in a study in which the psychobiotic potential of a probiotic formulation containing 2 × 10^9^ CFU/g of each of *Lactobacillus acidophilus, Lactobacillus casei, Bifidobacterium bifidum,* and *Lactobacillus fermentum* in patients suffering from multiple sclerosis was assessed by Kouchaki et al. (2017). Sixty patients with diagnosed multiple sclerosis took part in this randomized, double-blind, placebo-controlled trial for 12 weeks. Expanded disability status scale and mental health parameters were recorded at the baseline and 12 weeks after the intervention. The inflammation markers, oxidative stress biomarkers, and metabolic profiles were also analyzed. Compared with the placebo, probiotic intake improved the expanded disability status scale score and depression, anxiety, and stress scale. Changes in C-reactive protein, plasma nitric oxide metabolites, and malondialdehyde in the probiotic group were significantly different. Additionally, the consumption of probiotic formulation significantly decreased serum insulin levels. The psychobiotic potential of this specific formulation could attributed to the anti-inflammatory and anti-oxidative properties of probiotics, which prevents the production of free radicals and pro-inflammatory substances that promote the demyelination in the pathogenesis of multiple sclerosis [72]. Regarding the antioxidant effect of formulations and its role in exerting the psychobiotic potential, the effect of a probiotic formulation containing *Lactobacillus acidophilus, Lactobacillus casei*, and *Bifidobacterium bifidum* on symptoms of depression and metabolic status among patients with MDD was studied [71]. Forty patients diagnosed with MDD were randomized in a double-blind placebo-controlled clinical trial. The subjects were divided into two groups, where one received a probiotic capsule containing 2 × 10^9^ CFU/g of each of the three strains while the other group received a placebo for 8 weeks. Blood samples were taken at the beginning and at the end of the study. After the 8 weeks of treatment, the patients that received the probiotic formulation had significantly decreased the overall Beck Depression Index while the placebo did not. Additionally, the treatment group also exhibited higher total glutathione plasma levels, an antioxidant related to neuroinflammation in MDD.

## 5. A Practical Guide for Evaluating the Psychobiotic Potential

**Figure 3:**
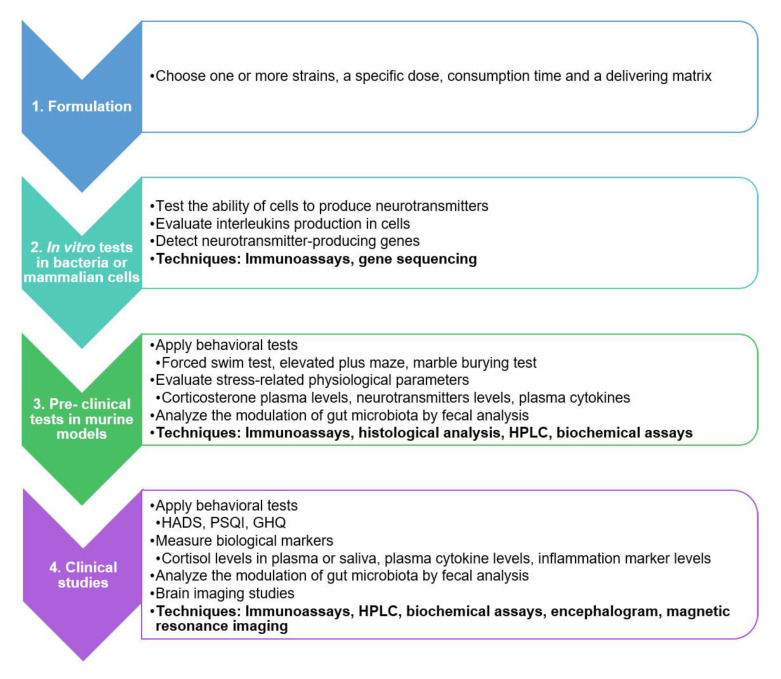
Proposed general methodology to evaluate psychobiotic potential of a microorganism or microbial formulations. HPLC, high performance liquid chromatography; HADS, Hospital Anxiety and Depression Scale; PSQI, Pittsburgh Sleep Quality Index; GHQ, General Health Questionnaire.

With the many characteristics that need to be considered to evaluate the psychobiotic potential, it could be perceived as too complicated and confusing. Therefore, a general practical guide is proposed in order to make the research job easier and less overwhelming. The guide is presented in Figure 3, which includes a step-by-step procedure, with techniques and trials ranging from in vitro assays to a series of tests in murine models and clinical trials with subjects, with the aim of detecting bacterial genes capable to produce neurotransmitters, identifying anti-inflammatory potential, and interleukin production, among other neuroactive metabolites. Additionally, biomarkers related to stress physiology can be measured, and more specialized tests can relate the taking of probiotics with the reduction of anxiety, depression, or some mental disorder symptoms. Some of the techniques are routinely applied in vitro, animal, and clinical studies. Nonetheless, we designed a logical sequence needed for a robust evaluation of psychobiotic potential of microorganisms or microbial formulations.

The first step should be to select a specific strain or formulation whose psychobiotic potential is sought to be assessed. In fact, any microorganism can be screened for its psychobiotic effect. However, the first criteria that the latter should meet is that the microorganism or microorganisms in question have already been identified as probiotic organisms. Furthermore, it is advised to perform a literature research beforehand to investigate whether the desired strain or strains or similar have already been studied for that matter and start from there. If not, the proposed methodology can be followed from the beginning. Afterwards, both the concentration and consumption time should be specified, as well as the delivering matrix, which could be a bacterial suspension, a pill, a fermented beverage or food, etc. Then, to perform a quick screening of those strains with intrinsic psychobiotic activity, in vitro tests can be carried out in order to test the ability of the bacteria to produce stress-related molecules, neurohormones, or other bioactive compounds, or modulate the production of the latter in a specific cell line. The most used tests for this matter include immunoassays or gene sequencing techniques. After the initial screening has been performed, a broader approach can be followed to evaluate the psychobiotic effects, but in vivo through pre-clinical trials using murine models. These tests could be used to assess any changes at either cognitive or physiological levels through behavioral tests that measure the animal response to stressful events. This response can also be evaluated directly by measuring stress-related physiological parameters at plasma, tissue, or biological fluid levels. Furthermore, through pre-clinical trials, a direct evaluation of the microbiota composition can be assessed at fecal level. Some of the most common evaluation techniques used in pre-clinical tests include immunoassays, histological analyses, and HPLC among other biochemical tests. Finally, a similar approach can be followed with a broader scope using human volunteers in clinical studies. This enables the researchers to perform more complex imaging studies such as encephalograms or MRI that give a better understanding of the brain activity.

## 6. Application of Potential Psychobiotic Strains on Fermented Foods and Beverages

Fermentation of food and beverages by probiotic strains, which are included in dietary practices, have been demonstrated to have potential health benefits not only by protecting the intestinal barrier, improving nutritional status, or limiting the growth of pathogens, but also by influencing brain health with mechanisms of action such as the production of neurotransmitters, direct activation of neural pathways between gut and brain, modulating neurotrophic chemicals, and showing analgesic properties [76]. A wide range of studies has reported different ways in which fermented foods or beverages directly influence mood and behavior or impact a variety of neurological disease symptoms through gut microbiota. For instance, a study in humans evaluated if fermented foods that contain probiotics such as yogurt, kefir, tempeh, kimchi, among others, were related to social anxiety in a population of young adults. Results demonstrated that fermented food consumption was negatively correlated with social anxiety, indicating that those who consume more fermented foods have lower social anxiety [77]. A summary of some other recent examples is presented in Table 2. It is worth noticing that due to the nature of fermented food, there are other ingredients present in the matrix. As far as this research went, no study was found where the interference or possible psychobiotic effect of these other ingredients was assessed. However, it is likely that they exert some effect on the gut-brain by themselves. That is why it would be advised to test the psychobiotic effect of fermented products without the microorganisms. For instance, by submitting the food to a thermal activation treatment prior the ingestion and studying its effect.

### 6.1. Dairy Products

Milk and dairy products are included in nearly every dietary guideline; fermented milk is the most regular formulation found to evaluate potential probiotic strains, being LAB the most frequent bacteria found in fermented dairy foods, either as starter cultures or as naturally occurring members of the raw material. These probiotic strains are widely associated with improved health outcomes [93]. Further, potential psychobiotic effects have been reported in a wide range of studies, focusing mainly on the lactobacilli and bifidobacteria activity. In this regard, *Lactobacillus casei* Shirota is one of the first strains in which psychobiotic potential was sought. Benton, William, and Brown measured the effect of milk fermented with *Lactobacillus casei Shirota* (1 × 10^8^ CFU/mL) on mood and cognition of subjects at baseline, and after 10 and 20 days of consumption with different tests. They found a general improvement of mood, though there were no relevant conclusions regarding memory measures [82]. This strain was also studied by Kato-Katoaka et al. (2016) in a fermented milk product, administered to students under academic stress. This study reported that the ingestion of 100 mL of fermented milk containing *Lactobacillus casei Shirota* (>1 × 10^9^ CFU/mL) for 8 weeks increased fecal serotonin levels compared to those of the placebo group. Moreover, the daily intake of fermented milk significantly reduced the total number of days experiencing physical symptoms (as a response to stress) and the rate of subjects experiencing these symptoms under stressful situations [57].

*Lactobacillus helveticus* has also been used to elaborate fermented milk to evaluate the cognitive function in subjects after its consumption. In this context, Chung et al. (2014) evaluated the cognitive function through neuropsychological and cognitive fatigue tests and measurements of the perceived stress scale (PSS), geriatric depression scale-short form (GDS-SF), brain-derived neurotrophic factor (BDNF), and whole blood viscosity (WBV) before and after the experiment. The administration of tablets (500, 1000, 2000 mg, or the placebo) of fermented milk powder with *Lactobacillus helveticus* IDCC3801 for 12 weeks in healthy older adults showed no significant effects for PSS, GDS-SF, BDNF, and WBV. However, the treatment improved cognitive tests compared to those of the placebo group [76]. In another study, healthy middle-aged adults received, during 8 weeks, 190 g of *Lactobacillus helveticus*-fermented milk drink containing a lactononadecapeptide to determine the effects on the cognitive function. This was evaluated through a Japanese version of the Repeatable Battery for the Assessment of Neuropsychological Status (RBANS) test. Results indicated a significant improvement in the total score, attention score, and delayed memory score of participants who received the milk drink compared to the placebo group [79].

Additionally, several probiotic combinations in fermented milk have shown potential psychobiotic effects. Akbari et al. (2016) developed a probiotic milk supplementation containing *Lactobacillus acidophilus, Lactobacillus casei, Bifidobacterium bifidum,* and *Lactobacillus fermentum* (2 × 10^9^ CFU/g for each). They administered 200 mL daily for 12 weeks to Alzheimer’s disease patients. Further, the mini-mental state examination (MMSE) score was recorded in all subjects before and after the treatment. Patients whom consumed the fermented milk showed a significant improvement in the MMSE score compared to the placebo group [82]. Another study assessed the neuroprotective effect (anti-inflammation) of a mixture of *Lactobacillus fermentum* LAB9, *Lactobacillus casei* LABPC (1 × 10^9^ CFU/0·2 mL), and cow milk in vitro and its attenuating memory deficit in mice using the Morris Water Maze Test. Mice were administered with treatments via oral gavage for 28 d daily; they were also injected (except for the control group) with lipopolysaccharides (LPS) intraperitoneally for four consecutive days. The mixture induced neuroprotection, associated with restoration of the cholinergic neurotransmission and attenuation of neuroinflammation. Additionally, there was an improvement in spatial learning and memory of LPS-induced mice. Moreover, in vitro, the results showed a decrease in nitrosative stress parameters [81].

Tillisch et al. (2013) investigated if the consumption of a fermented milk by healthy women altered brain intrinsic connectivity or responses to emotional attention tasks. The fermented milk contained *Bifidobacterium animalis* subsp *lactis* (1.25 × 10^10^ CFU)*, Streptococcus thermophilus*, *Lactobacillus bulgaricus* (1.2 × 10^9^ CFU)*,* and *Lactococcus lactis* subsp *lactis* (1.2 × 10^9^ CFU). After 4 weeks of chronic consumption of 125 g of the fermented milk, participants underwent fMRI. The formulation modulated the responsiveness of an extensive brain network and affected the brain regions’ activity that controls central processing of emotion and sensation [83].

Kefir and yogurt are also dairy beverages fermented by LAB strains, which have also been studied for their psychobiotic effects. Nishihira et al. (2014) studied the effect of yogurt fermented with *Lactobacillus gasseri* SBT2055 (≥5 × 10^8^ CFU) and *Bifidobacterium longum* SBT2928 (≥1 × 10^7^ CFU) on the immune function and mental and physical stress-induced disorders. Subjects consumed 100 g of yogurt daily for 12 weeks; natural killer (NK) cell activities and the levels of cortisol and adrenocorticotrophic hormone (ACTH), stress-induced hormones, were measured before and after consumption. Moreover, subjects answered the General Health Questionnaire-28. Yogurt enhanced the NK cell activity and reduced ACTH levels after the 12-week yogurt intake compared with their levels before yogurt intake. Interestingly, the tested yogurt suppressed serum cortisol release in younger males aged less than 65 years. Finally, data from the GHQ-28 demonstrated that the test yogurt contributed to the stress level reduction [92].

Furthermore, the effect of probiotic yogurt containing *Lactobacillus acidophilus* LA5 and *Bifidobacterium lactis* BB12 (1 × 10^7^ CFU) on mental health and HPA axis was analyzed in petrochemical workers. General health questionnaire (GHQ) and depression anxiety and stress scale (DASS) scores were used as parameters to measure mental health. Persons received 100 g of probiotic yogurt for 6 weeks. Blood samples were taken to measure serum levels of kynurenine, tryptophan, neuropeptide Y, cortisol, and ACTH. The probiotic yogurt administration had a positive effect on their mental health, but did not affect the HPA axis; there was a significant improvement in GHQ and DASS scores [91].

Recently, van de Wou et al. (2020) demonstrated that the fermented milk kefir can modulate specific aspects of the microbiota-gut-brain axis in mice. They provided two different kefirs to mice by oral gavage for 3 weeks. Several tests, including open-field test, stress-induced hyperthermia test, and forced swim test, among others, and measurements of the systemic immunity and serotonin levels in the gut, were assessed. Moreover, gut microbiota was studied by metagenomic sequencing of fecal matter and fecal metabolome analysis. The two kefirs affected both repetitive and reward-associated behavior in mice significantly. They also changed the composition of the host microbiota and differentially impacted systemic immunity and colonic serotonergic signaling. Besides, kefir consumption influenced specific gut microbial functional capacities, including GABA’s biosynthesis linked to an increased prevalence of *Lactobacillus reuteri* [89].

An approach with milk fermented with kefir grains was studied to improve cognitive and metabolic and/or cellular disorders in Alzheimer patients. For 90 days, patients consumed 2 mL/Kg daily of kefir; before and after the consumption, a cognitive assessment was evaluated with a battery of tests such as the Mini-Mental State Examination (MMSE) and immediate and delayed memory test, among others. Cytokine expression, systemic oxidative stress levels, and blood cell damage biomarkers were also measured. Favorable effects were observed on cognitive dysfunction (improving memory, language, executive functions, visual-spatial function, conceptualization, and abstraction abilities). Furthermore, there was a reduction of proinflammatory cytokines and systemic oxidative stress [88].

Butler et al. (2020) studied the impact of unpasteurized dairy products on the microbiota recently. During 12 weeks, participants consumed organic products including unpasteurized milk and dairy products. Before and after this, subjects provided fecal samples and completed self-report questionnaires such as Perceived Stress Scale (PSS), Hospital Anxiety and Depression Scale (HADS), Pittsburgh Sleep Quality Index (PSQI), and International Physical Activity Questionnaire (IPAQ). Stress and anxiety levels reduced significantly in those with higher baseline scores on the PSS and HADS-A., and there was an abundance of *Lactobacilli* and SCFAs observed in participants who consumed dairy products [90].

### 6.2. Soybean Products

Some approaches include using fermented soybean products to treat depression and cognitive impairment. In the first case, a study used fermented soy-based milk by *Lactobacillus brevis* FPA 3709 (1 × 10^6^ CFU/mL), a strain selected for its ability to produce GABA during cultivation with monosodium glutamate, and tested its anti-depressant activity in Sprague-Dawley (SD) rats. After 28 days of consumption of the black soybean fermented milk, rats were subjected to a forced swimming test. Results showed that the fermented soy-based milk enriched in GABA had an antidepressant effect similar to that of fluoxetine, a common antidepressant drug [80]. A recent study investigated *Lactobacillus plantarum* C29-fermented soybean’s efficacy and safety as a nutritional supplement for cognitive enhancement in patients with mild cognitive impairment (MCI). For 12 weeks, participants consumed 800 mg/day of a mixture of fermented soybean powder and *Lactobacillus plantarum* C29 (1.25 × 10^10^ CFU/g) freeze-dried powder. Computerized neurocognitive function tests were performed at baseline and repeated at 12 weeks; a change in the composite score of cognitive functions including attention, working memory, and verbal memory was observed. Changes in the microbiota were evaluated, observing that the *lactobacilli* population was significantly increased in the group that consumed the fermented product compared to the placebo group [86].

### 6.3. Other Fermented Products

One of the first approaches reported in the literature is the anti-stress and anti-fatigue effect of fermented rice bran in rats or mice. Both rats and mice consumed 1 g/kg/day of a hot water extract of fermented rice bran by Saccharomyces cerevisiae IFO 2346 for 2 weeks, and immobilized stress assay was performed on rats, then each organ was weighed and whole blood was obtained to measure changes in the adrenal, spleen, thymus and thyroid weights, lactate dehydrogenase (LDH), alkaline phosphatase (ALP), and cholesterol serum levels induced by stress. Besides, the anti-fatigue experiment and the swimming capacity were studied in mice. Fermented rice bran inhibited a major change in weight of the adrenal, thymus, and spleen on rats, and the swimming time of the mice consuming the fermented rice bran was enhanced significantly from that of the control group on day 15 [87]. 

A recent study investigated the neuroprotective and antioxidant potential of fermented *Laminaria japonica*, a food resource with reported bioactive potency. Subjects received 1.5 g/day of fermented *Laminaria japonica* for 6 weeks and performed various neuropsychological tests. Analyses of antioxidant function and inflammatory markers were also developed using serum samples. Subjects who consumed the fermented product improved the neuropsychological test scores, getting higher scores in the MMSE, numerical memory test, Raven test, and iconic memory, compared to the control group. In addition, fermented *Laminaria japonica* contained bioactive constituents such as GABA, and seemed to increase its antioxidant potency [85].

## 7. Future Tendencies and Conclusions

Depression and anxiety are mental disorders with a high prevalence worldwide that affect the quality of life of patients and impact the economy of entire regions. Effective treatments for both disorders are already in existence, but many of them have undesired secondary effects. Psychobiotics are a novel class of probiotics that possess anxiolytic and antidepressant properties in the host, accompanied by changes in their neural, emotional, and systemic states. Recent evidence suggests the HPA axis’s involvement, regulation of the immune system and inflammation, and the interaction between fermentation products with the host such as SCFAs and neuroactive compounds as possible action mechanisms behind the psychobiotic effect. A wide array of current studies has shown the potential psychobiotic effects that some bacteria strains and certain probiotic formulations possess against depression and anxiety in both human and murine models. Among those studies, a few have focused on assessing the psychobiotic potential of fermented products or bacteria formulations contained in a given food matrix.

The action mechanisms by which psychobiotic microorganisms exert their psychobiotic potential is not fully comprehended yet, and there are some gaps in its understanding. For instance, there are no concluding studies that accurately describe how the microbiota communicates with the brain through the vagus nerve, only those investigations that claim its involvement, but not precisely how. Moreover, the specific metabolic routes regarding the production of neuroactive compounds in most psychobiotic strains have not been completely elucidated or are only partially described for some strains. However, one of the biggest gaps is the lack of systemic studies that evaluate the psychobiotic effect of strains from in vitro studies all the way to clinical studies. This type of studies that contemplate the psychobiotic extent at different levels would allow a better understanding not only of the potential itself, but also of the mechanism behind it.

An increasing number of rodent and human studies report the psychobiotic potential of microorganisms, altering the gut microbiota, improving cognitive function, and modulating anxiety and stress levels. However, due to the complexity of the gut-brain-microbiota axis, elucidating the specific mechanisms by which bacteria and yeast strains exert their activity and identifying a systemic procedure to assess the psychobiotic effects of a determined strain, formulation, or food product, remains a challenge. In this sense, there is a wide variety of experiments and results, and thus a lack of consensus in general. In fact, to our knowledge, there had been no systematic study of the evaluation of potential psychobiotics according to their mechanism of action or experimental methodology.

Finally, we are currently facing one of the biggest challenges in modern history with the COVID-19 outbreak. Not only because of the highly infectious virus, but also because of the threat this pandemic poses to everyone’s mental health. That is why the consumption of psychobiotics could be seen as a viable option to both look after and even restore any damages done to our mental health during this unprecedented time without undesired secondary effects. Especially because psychobiotics belong to microbiota naturally found in the intestinal tract, they may offer a lower risk of allergies and less dependence than psychotropic drugs [94].

## Figures and Tables

**Table 1:** Bacterial strains and formulations with psychobiotic potential effect.

Bacteria	Model	Dose	Findings	Possible Mechanism	Reference
Single-strain probiotic					
*Bacillus coagulans* MTCC 5856	Clinical trial	2 billion spores	Robust efficacy for the treatment of patients experiencing IBS symptoms with major depressive disorder.	Production of SCFAsand antimicrobial and anti-inflammatory substances.	[52]
*Bifidobacterium longum* 1714	Clinical trial	1 × 10^9^ CFU/day	Reduced stress and improved memory.	Brain-Derived Neurotrophic Factor (BDNF) synthesis through vagal activity.	[53]
*Bifidobacterium longum* NCC3001	Clinical trial	1 × 10^10^ CFU/g	Reduction in depression scores on Hospital Anxiety and Depression Scale and reduced responses to negative emotional stimuli in multiple brain areas.	Release of neuroactive compounds through vagal signaling as well as BDNF regulation.	[54]
*Clostridium butyricum* MIYAIRI 588	Clinical trial	60 mg/day	In combination with antidepressants, is effective in the treatment of treatment-resistant major depressive disorder.	Regulation of proinflammatory agents.	[55]
*Lactobacillus casei* Shirota	Clinical trial	1 × 10^9^ over 8 weeks	Decrease in the cognitive state anxiety scores, somatic state, and perceived stress scale.		[56]
*Lactobacillus casei* Shirota	Clinical trial	100 mL of a fermented beverage containing more than 1 × 10^9^ CFU/mL/day	Salivary cortisol and plasma L-tryptophan levels were increased in the placebo group, while the experimental group had higher fecal serotonin levels. Lower rate of subjects experiencing common abdominal and cold symptoms, and total number of days experiencing these physical symptoms.	Hypothalamic-pituitary-adrenal (HPA) axis modulation and promotion of serotonin synthesis.	[57]
*Lactobacillus gasseri* CP2305	Clinical trial	1 × 10^10^ CFU	Stress-associated behaviors were improved, as well as the sleeping quality. The parabiotic administration also prevented increases in basal salivary cortisol release and expression of stress-responsive microRNAs.	Regulation of inflammation mechanisms.	[58]
*Lactobacillus casei* Shirota	Clinical trial and in vivo murine model	Milk fermented with 1 × 10^9^ CFU/mL	Increases in salivary cortisol levels and incidence rate of physical symptoms were significantly suppressed. In rats, water avoidance stress-induced increases in plasma corticosterone were suppressed, and the number of corticotrophin releasing factor-expressing cells in the paraventricular nucleus was reduced.	HPA axis regulation.	[59]
*Bifidobacterium breve* 1205	In vivo murine model	1 × 10^9^ CFU/mL	Reduced anxiety in the marble-burying test and induced lower anxiety in the elevated plus maze.	Immune system regulation and gut hormones secretion.	[60]
*Bifidobacterium breve CCFM1025*	In vivo murine model	0.1 mL/10 g body weight daily at 1 × 10^9^ CFU/mL	Reduced depression and anxiety behaviors. The hyperactive HPA response and inflammation were also alleviated. Expression of the brain-derived neurotrophic factor was increased, while chronic stress was restored.	Capacity of SCFAs to improve serotonin levels, and regulation of the HPA axis and BDNF synthesis.	[61]
*Bifidobacterium infantis* 35624	In vivo murine model	1 × 10^10^ live bacterial cells/100 mL drinking/day	Normalization of the immune response, reversal of behavioral deficits, and restoration of basal noradrenaline concentrations in the brain.	Anti-inflammatory properties.	[62]
*Bifidobacterium longum* 1714	In vivo murine model	1 × 10^9^ CFU/mL	Reduced anxiety in the marble-burying test; decreased stress-induced hyperthermia, lower anxiety in the elevated plus maze, and antidepressant-like behavior in the tail suspension test.	Immune system regulation and gut hormones secretion.	[60]
*Faecalibacterium prausnitzii* ATCC 27766	In vivo murine model	1 × 10^9^ CFU by oral gavage	Preventive and therapeutic effects on depression and anxiety behavior, higher levels of SCFAs in the cecum, and higher levels of cytokines interleukin-10 in the plasma. Corticosterone, C-reaction protein, and Interleukin-6 levels were normalized.	SCFAs synthesis, immune system stimulation, and HPA axis regulation.	[63]
*Lactobacillus helveticus* NS8	In vivo murine model	1 × 10^9^ CFU/mL in drinking water	The anxiolytic effect in hyperammonia-treated rats was associated with a reduction in hippocampal serotonin 5-HTP levels.	Downregulation of inflammation and serotonin metabolism.	[64]
*Lactobacillus plantarum* ATCC 8014	In vivo murine model	Bacterial suspensions at 1 × 10^7^ CFU/mL	Favorable effects on oxidative markers of the blood and amygdala, as well as on concentrations of amygdala serotonin and brain-derived neurotrophic factor (BDNF). Beneficial effects were observed on the elevated plus maze and forced swimming tests.	HPA axis downregulation due to oxidative stress reduction.	[65]
*Lactobacillus plantarum* PS128	In vivo murine model	1 × 10^9^ CFU/mouse/day by gavage	Anxiety behavior in naïve adult mice was reduced, whereas the depressive behaviors were reduced in early life stressed mice. Early life stressed mice-induced elevation of serum corticosterone decreased, inflammatory cytokine levels reduced, and anti-inflammatory cytokine levels increased. Dopamine levels in rose in both groups, whereas serotonin level was increased in the naïve adult mice.	HPA axis regulation, modulation of the immune system, and synthesis of neuroactive compounds.	[66]
*Lactobacillus rhamnosus* JB-1	In vivo murine model	1 × 10^9^ CFU	Reduction of the content of corticosterone and restricted behaviors associated with depression and anxiety. Neurochemical and behavioral effects were absent in mice after vagotomy.	GABA synthesis and regulation of the HPA axis.	[67]
*Lactobacillus rhamnosus* GG	In vivo murine model	1 × 10^9^ CFU	Attenuated OCD-like behavior induction: Increased perseverative open-field locomotion, stereotypic turning, and marble burying.	Brain serotogenic system.	[68]
Formulations (multi-strain probiotics					
*Lactobacillus helveticus* R0052 and *Bifidofacterium longum* R0175	Clinical trial	1 × 10^9^ CFU/5 g	Significant decrease in Beck Depression Inventory (BDI) score. The kynurenine/tryptophan ratio decreased significantly in the probiotic group, while the tryptophan/isoleucine ratio increased.	Modulation of neurotransmitters synthesis and metabolism.	[69]
*Bifidobacterium bifidum* W23*, Bifidobacterium lactis* W52, *Lactobacillus acidophilus* W37, *Lactobacillus brevis* W63, *Lactobacillus casei* W56, *Lactobacillus salivarius* W24, *and Lactococcus lactis* (W19 and W58)	Clinical trial	2.5 × 10^9^ CFU/g	Reduced overall cognitive reactivity to sad mood.		[70]
*Lactobacillus acidophilus, Lactobacillus casei* and *Bifidobacterium bifidum*	Clinical trial	2 × 10^9^ CFU/g of each strain	Significant improvement in depression scores on the Beck Depression Inventory, and reduced serum insulin and increased plasma total glutathione levels.	Decreased oxidative stress.	[71]
*Lactobacillus acidophilus, Lactobacillus casei, Bifidobacterium bifidum* and *Lactobacillus fermentum*	Clinical trial	1 capsule containing 2 × 10^9^ CFU/g daily for 12 weeks	Improved expanded disability status scale, Beck Depression Inventory, general health questionnaire, and depression anxiety and stress scale. Changes in C-reactive protein, plasma nitric oxide metabolites, and malondialdehyde.		[72]
*Lactobacillus helveticus * R0052 and *Bifidobacterium longum* R0175	Clinical trial and in vivo murine model	3 × 10^9^ CFU/stick daily for 30 days	Reduction of anxiety-like behavior in rats, alleviation of psychological distress in volunteers, and a reduction in urinary free cortisol levels.	HPA axis regulation.	[73]
*Bifidobacterium longum* subsp. *infantis* E41 and *Bifidobacterium breve* M2CF22M7	In vivo murine model	1 × 10^9^ CFU	Reduction in depressive behaviors of mice as well as increased the level of 5-hydroxytryptamine and brain-derived neurotrophic factor concentration in the brain.	Downregulation of HPA axis, and changes in levels of 5-HTP through SCFAs synthesis.	[64]
*Lactobacillus helveticus* R0052 and *Bifidobacterium longum* R0175	In vivo murine model	1 × 10^9^ CFU in 0.9% NaCl	Attenuated HPA axis and autonomic nervous system activities in response to water avoidance stress, and reduced cFos protein expression in different brain areas. Probiotic pretreatment prevented hippocampal neurogenesis and expression changes in hypothalamic genes involved in synaptic plasticity.	Changes in brain plasticity due to BDNF production, and HPA axis regulation.	[18])
*Lactobacillus plantarum* 90sk and *Bifidobacterium adolescentis* 150	In vivo murine model	1 dose (0.5 mL) of the mixture of 1 × 10^8^ CFU *L. plantarum* 90sk and 1 × 10^7^ CFU *B. adolescentis* 150	Reduced depressive-like behavior in the forced swimming test; the effect was similar to that of fluoxetine.	GABA production.	[74]

CFU: Colony Forming Units, IBS: Irritable Bowel Syndrome, SCFAs: Short Chain Fatty Acids, BDNF: Brain-Derived Neurotrophic Factor, HPA: Hypothalamic-pituitary-adrenal, HTP: hydroxytryptamine.

**Table 2:** Potential psychobiotic applications in fermented foods products.

Food Product	Strain(s)	Model	Doses	Psychobiotic Effects	Reference
Black soybean milk	*Lactobacillus helveticus IDCC3801*	Clinical trial	500, 1000, or 2000 mg of tablets of skim milk powder fermented for 12 weeks	Improvements in cognitive function in healthy old adults	[78]
	*Lactobacillus helveticus*	Clinical trial	190 g of fermented milk once a day for 8 weeks	Improved cognitive function in healthy middle-aged adults	[79]
	*Lactobacillus* *brevis FPA 3709*	In vivo murine model	35 mg/kg or 70 mg/kg body weight by oral gavage for 28 days	Antidepressant effect without side effects in rat models	[80]
	*Lactobacillus fermentum* LAB9 or *Lactobacillus casei* LABPC	In vivo murine model	Daily administration by oral gavage for 28 days	There was a restoration of cholinergic neurotransmission and attenuation of neuroinflammation in mice	[81]
Fermented milk	*Lactobacillus gasseri CP2305*	Clinical trial	190 g of fermented milk once a day for 5 weeks	Improved sleep quality and alleviated stress-associated symptoms in healthy students	[58]
	*Lactobacillus casei Shirota*	Clinical trial	100 mL of fermented milk once a day for 8 weeks	Increased fecal serotonin levels and reduced physical symptoms in healthy subjects when exposed to stressful situations	[57]
		Clinical trial	65 mL of probiotic-containing milk drink for 3 weeks	Improved the mood of adults whose mood was initially poor/depressive	[82]
	*Bifidobacterium animalis* subsp *lactis I-2494, Streptococcus thermophilus I-1630,* * Lactobacillus bulgaricus I-1632 and I-1519, and Lactococcus lactis* subsp. *lactis I-1631*	Clinical trial	125 g of fermented milk twice daily for 4 weeks	Affected the activity of brain regions that control central processing of emotion and sensation in healthy women	[83]
	*Lactobacillus acidophilus, Lactobacillus casei, Bifidobacterium bifidum, and Lactobacillus fermentum*	Clinical trial	200 mL/day for 12 weeks	Positively affected cognitive function and some metabolic statuses in Alzheimer’s disease patients (60–95 years old)	[84]
	*Lactobacillus casei* Shirota YIT 9029	Clinical trial and In vivo murine model	100 mL of fermented milk once a day for 8 weeks	Physical symptoms of stressed subjects were reduced	[59]
Fermented *Laminaria japonica*	*Lactobacillus brevis* BJ20	Clinical trial	1.5 g/day of fermented *Laminaria japonica* for 6 weeks	Provided a protective mechanism against cognitive impairment associated with dementia in elderly	[85]
Fermented soybean	*Lactobacillus plantarum* C29	Clinical trial	800 mg/day for 12 weeks	Improved cognitive function in individuals with Mild Cognitive Impairment	[86]
Fermented rice bran	*Saccharomyces cerevisae* IFO 2346	In vivo murine model	1 g/kg/day of a hot water extract of fermented rice bran	Demonstrated anti-stress and anti-fatigue effects in rats and mice	[87]
Kefir	*Acetobacter* * aceti, Acetobacter* sp.*, Lactobacillus delbrueckii,* * Lactobacillus fermentum, Lactobacillus fructivorans, Enterococcus faecium, Leuconostoc spp., Lactobacillus kefiranofaciens, Candida famata,* and *Candida krusei*	Clinical trial	2 mL/Kg/daily for 90 days	Improvement in memory, visual-spatial/abstraction abilities, and executive/language functions	[88]
	*Lactobacillus reuteri*	In vivo murine model	Daily administration 1 h by oral gavage for 3 weeks	Increased capacity of GABA production in the gut microbiota of mouse	[89]
Unpasteurized milk and dairy products	*Lactobacilli*	Clinical trial	Free consumption before and after 12 weeks	Decreased stress and anxiety scores in adults	[90]
Yogur	*Lactobacillus acidophilus* LA5 and *Bifidobacterium lactis* BB12	Clinical trial	100 g once a day for 6 weeks	Improvement of adults in depression anxiety and stress scale scores	[91]
	*Lactobacillus gasseri* SBT2055 *and Bifidobacterium longum* SBT2928	Clinical trial	100 g once a day for 12 weeks	Levels of the stress-induced hormone adrenocorticotrophic hormone significantly decreased in adults	[92]

## Supplementary Materials

The following are available online at https://www.mdpi.com/2072-6643/12/12/3896/s1, Table S1: Studies applying different tests to evaluate the psychobiotic potential of a strain, formulation or food product.
