# Supplementary material for: Psychobiotics: Mechanisms of Action, Evaluation Methods and Effectiveness in Applications with Food Products
Source: Nutrients. 2020 Dec 19;12(12):3896. doi: 10.3390/nu12123896 (PMC7767237; doi:10.3390/nu12123896)
Supplement: Supplementary file 1 [file nutrients-12-03896-s001.pdf]

Table S1. Studies applying different tests to evaluate the psychobiotic potential of a strain, formulation or food product.

Type of study	Experimental subject	Type of test	Test	References*
Preclinical	Rats or mice	Behavioral	Stress-induced hyperthermia	(Savignac, Kiely, Dinan, & Cryan, 2014; Bravo *et al.*, 2011; van de Wou *et al.*, 2020)
			Elevated plus maze	(Savignac, Kiely, Dinan, & Cryan, 2014; Hao, Wang, Guo, & Liu, 2019; Luo *et al.*, 2014; Morshedi *et al.*, 2018; Liu *et al.*, 2015; Bravo *et al.*, 2011; Tian, Wang, Zhao, Zhang, & Chen, 2019; Leclercq *et al.*, 2017; van de Wou *et al.*, 2020)
			Open field	(Savignac, Kiely, Dinan, & Cryan, 2014; Hao, Wang, Guo, & Liu 2019; Liu *et al.*, 2015; Kantak, Bobrow, & Nyby, 2014; Tian, Wang, Zhao, Zhang, & Chen, 2019; Leclercq *et al.*, 2017; van de Wou *et al.*, 2020)
			Forced swim test	(Savignac, Kiely, Dinan, & Cryan, 2014; Desbonnet *et al.*, 2010; Hao, Wang, Guo, & Liu 2019; Morshedi *et al.*, 2018; Liu *et al.*, 2015; Tian, Wang, Zhao, Zhang, & Chen, 2019; Yunes *et al.*, 2019; van de Wou *et al.*, 2020; Ko, Lin and Tsai, 2013; Bravo *et al.*, 2011; Ko, Lin, & Tsai, 2013)
			Sucrose preference test	(Liu *et al.*, 2015; Tian, Wang, Zhao, Zhang, & Chen, 2019; van de Wou *et al.*, 2020)
		Biologic studies	Corticosterone levels	(Savignac, Kiely, Dinan, & Cryan, 2014; Tian *et al.*, 2020; Hao, Wang, Guo, & Liu 2019; Morshedi *et al.*, 2018; Takada *et al.*, 2016; Liu *et al.*, 2015; Bravo *et al.*, 2011; Desbonnet *et al.*, 2010; Ait-Belgnaoui et al, 2014; Takada *et al.*, 2016; van de Wou *et al.*, 2020)
			Cytokines level	(Leclercq *et al.*, 2017; Tian *et al.*, 2020; Desbonnet *et al.*, 2010; Hao, Wang, Guo, & Liu 2019; Luo *et al.*, 2014; Musa *et al.*, 2017; van de Wou *et al.*, 2020)
			SCFAs content	(Tian *et al.*, 2020; Hao, Wang, Guo, & Liu 2019; Tian, Wang, Zhao, Zhang, & Chen, 2019; van de Wou *et al.*, 2020)
			Serotonin levels	(Yano *et al.*, 2015; Clarke et al., 2012; Tian *et al.*, 2020; Desbonnet *et al.*, 2010; Luo *et al.*, 2014;).
	In vitro	Bacterial	GABA production	(Siragusa *et al.*, 2007; Valenzuela *et al*. 2019; Yunes *et al.*, 2016; Cataldo *et al.*, 2020; Ko, Lin, & Tsai, 2013)
		Mammal cell line	Serotonin production	(Tian *et al.*, 2020; Tian, Wang, Zhao, Zhang, & Chen, 2019; Musa *et al.*, 2017)
Clinical	Humans	Behavioral	Hospital Anxiety and Depression scale	(Pinto-Sanchez *et al.*, 2017; Kato-Kataoka *et al.*, 2016; Nishida *et al.*, 2017; Messaoudi *et al.*, 2010; Butler *et al.*, 2020)
			General Health Questionnaire	(Nishida *et al.*, 2017; Kouchaki *et al.*, 2017; Nishihira *et al.*, 2014; Mohammadi *et al.*, 2015)
			Perceived stress scale	(Allen *et al.*, 2016; Adikari, Appukutty, & Kuan, 2019; Messaoudi *et al.*, 2010; Chung et al., 2014; Butler *et al.*, 2020)
			Pittsburgh sleep quality index	(Nishida *et al.*, 2017; Butler *et al.*, 2020)
			Mini-mental state examination	(Akbari *et al.*, 2016; Ton *et al.*, 2020; Reid, Ryu, Kim, & Jeon, 2018)
		Biologic markers	Cortisol levels	(Andersson *et al.*, 2016; Kato-Kataoka *et al.*, 2016; Takada *et al.*, 2016; Nishida *et al.*, 2017; Messaoudi *et al.*, 2010; Nishihira *et al.*, 2014; Mohammadi *et al.*, 2015)
			Tryptophan blood levels	(Kato-Kataoka *et al.*, 2016; Kazemi, Noorbala, Azam, Eskandari, & Djafarian, 2018; Mohammadi *et al.*, 2015)
			Inflammation markers levels	(Majeed, Nagabhushanam, Arumugam, Majeed, & Ali, 2018; Pinto-Sanchez *et al.*, 2017; Nishihira *et al.*, 2014; Ton *et al.*, 2020)
			Oxidative stress markers	(Akkasheh *et al.*, 2016; Kouchaki *et al.*, 2017; Akbari *et al.*, 2016; Ton *et al.*, 2020; Reid, Ryu, Kim, & Jeon, 2018)

	Neurotransmitter levels	(Pinto-Sanchez *et al*., 2017; Kato-Kataoka *et al*., 2016)
Imaging	MRI	(Pinto-Sanchez *et al*., 2017; Tillisch *et al*., 2013)
	Encephalogram	(Allen *et al*., 2016)

*Detailed information regarding references is shown in the main manuscript. MRI: Magnetic Resonance Imaging.
